# Rosmarinic acid prevents post-operative abdominal adhesions in a rat model

**DOI:** 10.1038/s41598-022-22000-x

**Published:** 2022-11-03

**Authors:** Ali Kakanezhadi, Mehrdad Rezaei, Abbas Raisi, Omid Dezfoulian, Farshid Davoodi, Hassan Ahmadvand

**Affiliations:** 1 Department of Clinical Sciences, Faculty of Veterinary Medicine, Lorestan University, Khorramabad, Iran; 2 Department of Pathobiology, Faculty of Veterinary Medicine, Lorestan University, Khorramabad, Iran; 3 Department of Surgery and Diagnostic Imaging, Faculty of Veterinary Medicine, Urmia University, Urmia, Iran; 4 Medicinal Plants and Natural Products Research Center, Hamadan University of Medical Sciences, Hamadan, Iran

**Keywords:** Biomarkers, Experimental models of disease, Cytokines, Biochemistry, Immunochemistry

## Abstract

This study aims to determine the effects of rosmarinic acid which involved the mechanisms to decrease the postoperative peritoneal adhesion formation in rats. Various incisions and removing a 1 × 1 cm piece of peritoneum was used to induce the peritoneal adhesions. Experimental groups were as follows: 1—Sham group. 2—Control group: Peritoneal adhesions were induced and no treatments were performed. 3—Treatment groups: Following inducing peritoneal adhesions, animals received rosmarinic acid with 50 and 70 mg/kg dosage, respectively. Macroscopic examination of adhesions indicated that adhesion bands were reduced in both treatment groups compared to the control group. Moreover, the adhesion score was decreased in both treatment groups on day 14. Inflammation and fibroblast proliferation were both reduced in the treatment groups on day 14. TGF-β1, TNF-α, and VEGF were all evaluated by western blot and immunohistochemistry on days 3 and 14. Treatment groups reduced inflammatory cytokines on days 3 and 14. The treatment group with a 70 mg/kg dosage decreased TGF-β1 and TNF-α levels more than the other treatment group. The administration of rosmarinic acid significantly reduced MDA and increased CAT levels. In conclusion, the rosmarinic acid was effective to reduce the adhesion bands, inflammatory cytokines, angiogenesis, and oxidative stress.

## Introduction

Peritoneal adhesions are fibrous connections formed in the damaged region of the peritoneum as a result of different events, including surgical operations^1^. With a prevalence of 66 percent, peritoneal adhesion syndrome produces various ailments, including abdominal discomfort, infertility in women, and intestinal blockage^2^. Peritoneal adhesions are common after abdominal surgery, with a reported prevalence of 93 percent^3^. Different events are effective to form the adhesions, including coagulation, inflammation, and fibrinolysis through the healing of the damaged area. Previous research assessed multiple substances comprising the drugs and barriers to impede the peritoneal adhesions^2^.

Inflammatory cells, such as lymphocytes, macrophages, and neutrophils penetrate the wound site after peritoneal damage in terms of the activation of inflammatory and coagulation pathways^4^. The number of polymorphonuclear neutrophils increases in the first two days following damage, followed by the amount of macrophages five or six days later^3^. Interleukin-1 (IL-1), tumor necrosis factor-alpha (TNF-α), transforming growth factor β (TGF-β), Interleukin-6 (IL-6), and other adhesion-associated cytokines are all secreted by macrophages^5,6^. Macrophages promote secretion leakage and adhesion by producing oxidative stress, which results in lipid peroxidation and cell membrane cytolysis^4,7^. At the same time, fibrinogen in plasma is converted to fibrin and coagulates with platelets once thrombin activates the coagulation process^2,8^. In situations with low oxygen pressure during the inflammatory process, angiogenesis starts by attacking the endothelial cells utilizing the fibrinolytic system^7^. Adhesive fibroblasts cause the capillary formation and increase vascular endothelial growth factor (VEGF) levels^7^. Primary fibrinolysis within five to seven days after injury is critical for adhesion prevention^2^. Collagen production, as well as the creation and structure of the extracellular matrix, might result in persistent adhesion, therefore avoidance is critical at this time^2,4^.

The ester of caffeic acid with 3,4-dihydroxy phenyl lactic acid is rosmarinic acid (RA). This phenolic chemical is found in the Boraginaceae family and Nepetoideae subfamily of plants. Anti-cancer, antioxidant, anti-aging, anti-inflammatory, anti-bacterial, anti-diabetic, and anti-allergic characteristics are all found in RA^9^. By enhancing insulin sensitivity, RA has hypoglycemic effects^10^. In the colons of cancer-stricken animals, RA reduced tumor formation^11^. In prostate cancer cell lines, RA accelerates apoptosis and the cell cycle^12^. TNF-α, cyclooxygenase-2 (COX-2), and IL-6 levels are reduced by RA, which also modifies the expression of p65 and blocks its transit from the cytosol to the nucleus^13,14^. In addition to its antibacterial effects against the *Staphylococcus aureus* and by increasing the effectiveness of antibiotics, RA has inhibitory effects on *Escherichia coli* K-12 growth^15,16^. RA decreases IL-8 distribution from endothelial cells and the expression of VEGF^17,18^. It prevents cardiac hypertrophy and interferes with various stages of angiogenesis. RA increases Superoxide dismutase (SOD), Catalase (CAT), and Glutathione peroxidase (GPx) activity and reduces lipid peroxidation, reactive oxygen species (ROS) generation, and proinflammatory mediators^9,19–21^. RA lowers oxidative stress indicators and protects liver cells from ischemia injury. Moreover, following spinal cord damage in rats, RA dramatically lowered oxidative stress^22^. By limiting the expression of α-smooth muscle actin (α-SMA) and the conversion of transforming growth factor β1 (TGF-β1)^23,24^ evidence shows that RA may diminish the degree of hepatic fibrosis and suppress the profibrotic response. By the changes in ERK11/2 signaling, RA is beneficial in alleviating depression^25^. P-tau formation is suppressed by RA, which may restore aberrant alterations generated by chaperones^26^. Tubular necrosis, urea, creatinine, and tubular epithelial degeneration are all reduced by RA^27,28^. TGF-β, IL-1 β, IL-6, TNF-α, and VEGF levels were all lower after RA treatment in hepatocellular cancer^29^. RA inhibits LPS-induced proinflammatory cytokine activation, including IL-10, IL-6, IL-1 β, INF-β, MCP-1, iNOS, and NF-κB^9^. In mice with arthritis, RA lowered inflammation, whereas in animals with nerve damage, it reduced prostaglandin E2 (PGE-2) and inflammatory markers in the spinal cord^30,31^.

This research was conducted to evaluate the preventive benefits of RA on postoperative peritoneal adhesion based on the beneficial qualities of RA and its influence on inflammatory markers and oxidative stress.

## Results

### Macroscopic observations showed that rosmarinic acid reduced postoperative peritoneal adhesion

**Figure 1:**
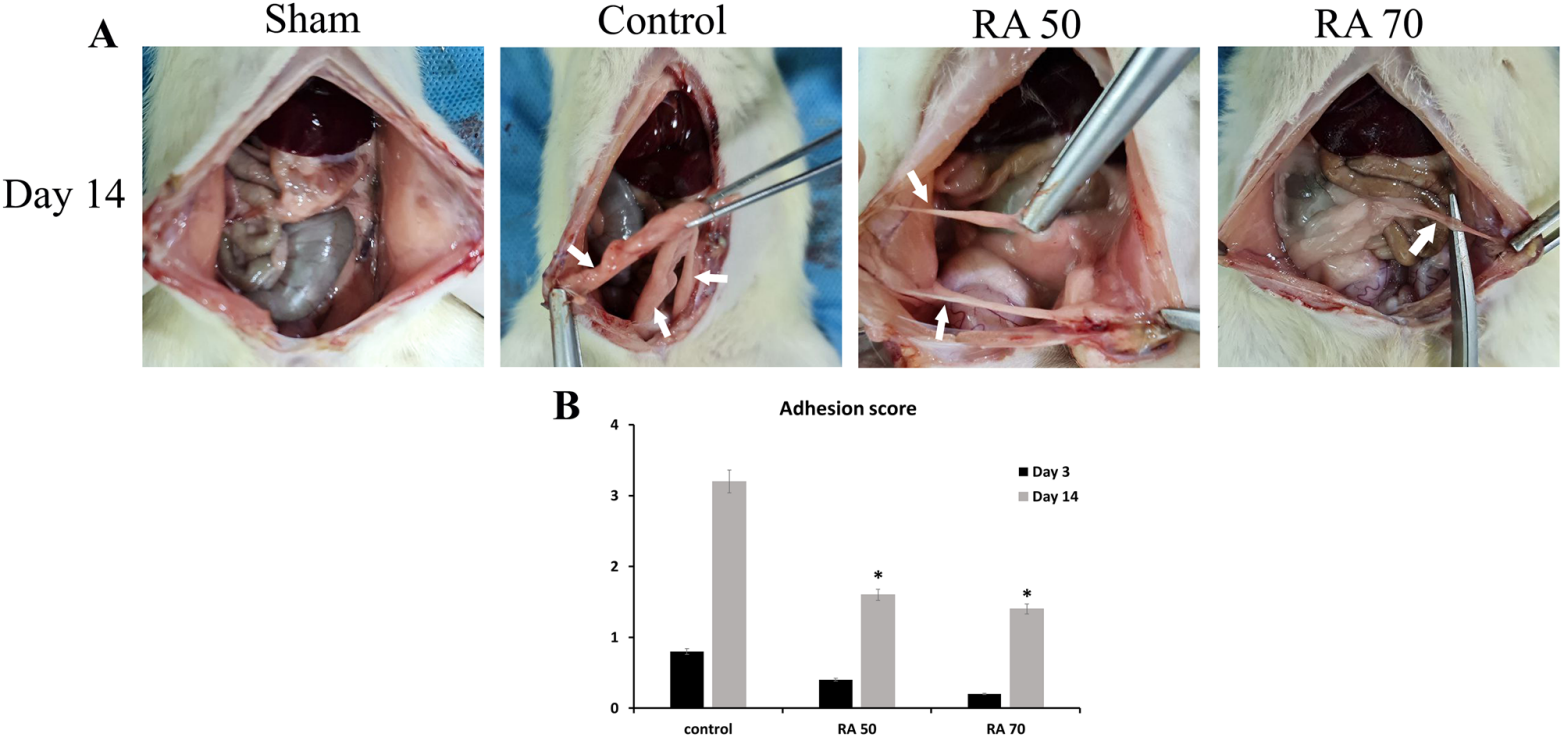
Macroscopic evaluation of adhesion bands in different experimental groups of the study on day 14. (**A**) The diagram shows the adhesion score by the method of Nair et al. (**B**) * indicates a significant difference with the control group.

Rats were sacrificed on days 3 and 14 following surgery to assess and monitor adhesion scores. The peritoneum and other components of visceral organs were shown to have adhesion bands, and rats showed variable degrees of adhesion (Fig. 1A). Figure 1B displays the mean adhesion scores of rats using the scoring system of Nair et al. in each of the groups on day 14 following surgery. Rats in the RA50 and RA70 groups had substantially lower scores than the control group (p < 0.05), as shown in Fig. 1B. On the 14th day following surgery, there was no significant difference in the mean scores of the therapy groups. On the third postoperative day, there was no significant difference among the groups (p > 0/05). Table 1 provides the percentage of rats which revealed adhesion bands in each group and visceral organs in which bands were attached.

**Table 1:**
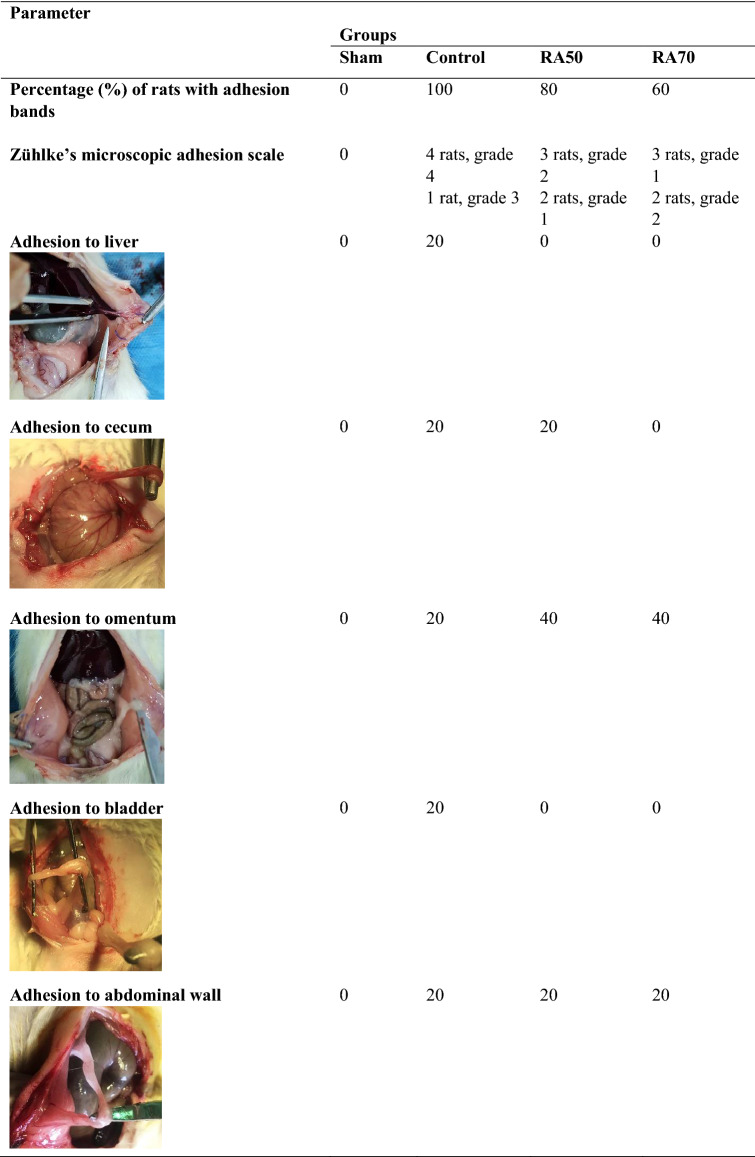
Percentage of rats with adhesion bands, Zühlke’s microscopic adhesion scale, and visceral organs in which bands were attached.

In the control group, rats developed more than one adhesion band in each group that was attached to various organs.

### Rosmarinic acid treatment reduced fibroblast proliferation and inflammation

**Figure 2:**
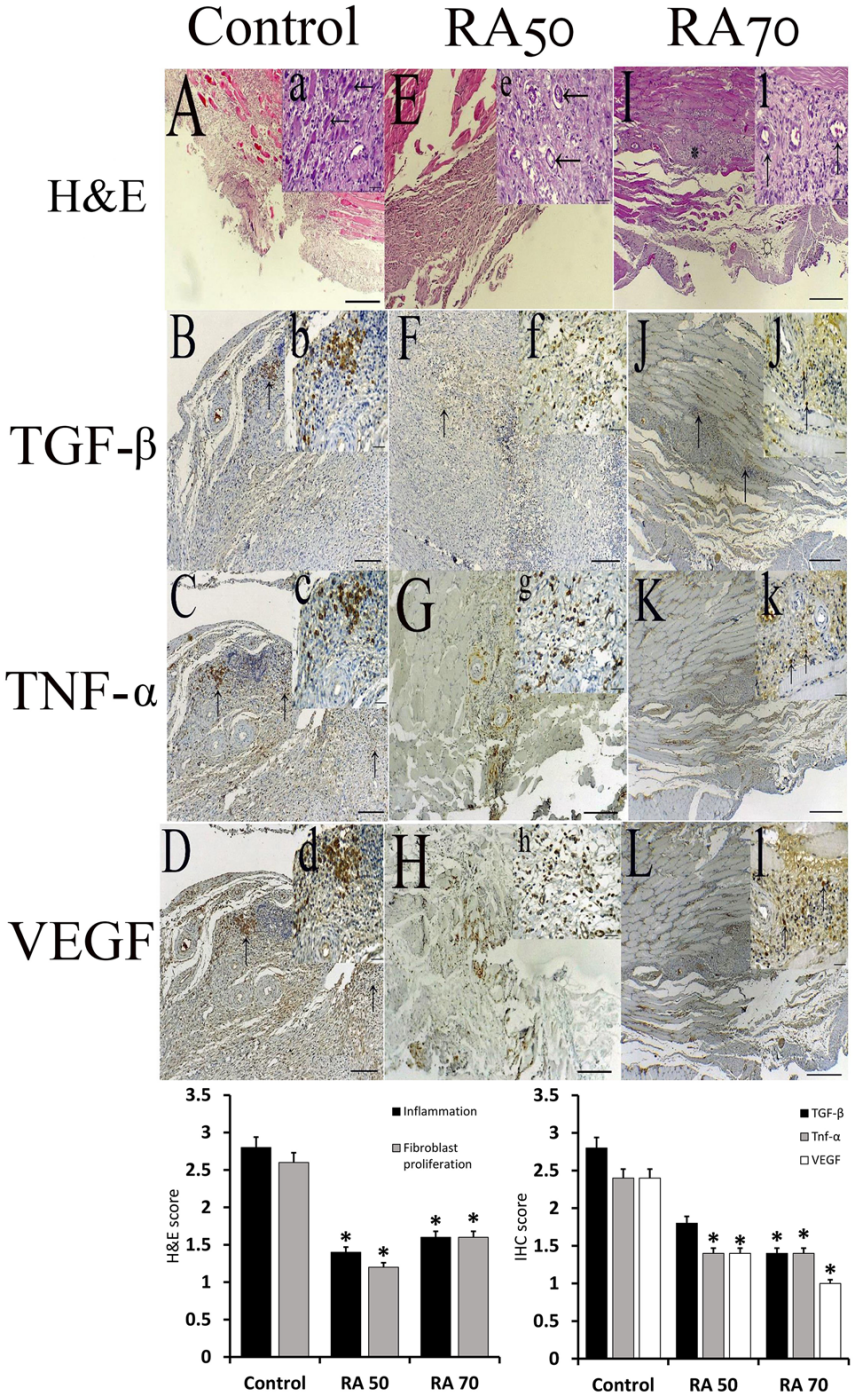
Control group{**A–D**}. (**A, a**): parietal peritoneum; Visceral cavity aspect: massive connective tissue fibroblast proliferation. A large population of fibroblasts and mononuclear inflammatory cells are infiltrated throughout the lesion, which stretched to the middle layers of striated muscles. (**A**). Ineffectual regeneration is demonstrated by bizarre multinucleate muscle giant cells (arrows) inset; (**a**), H&E. (**B, b**): immunoexpression of TGF-β1 in accumulated inflammatory cells (arrow) (**B**). Intensive cytoplasmic inset; (**b**). (**C, c**): Immunopositive staining of TNF-a in both accumulated and diffuse states (arrows) (**C**). Strongly cytoplasmic staining inset; (**c**). (**D, d**): immunolabeling of VEGF in both aggregated and diffused forms (arrows) (**D**). Strong cytoplasmic staining of inflammatory cells inset; (**d**). A–D = 400 µm & a–d = 40 µm. Rosmarinic 50 {**E–H**} (**E, e**): The fibroblasts and inflammatory cells, mainly mononuclear cells are limited at the margin of striated muscles of parietal peritoneum. (**E**). Dense collagen fibers with thick-walled microvessels are embedded at the center of the lesion (arrows) inset; (**e**). H&E (**F, f**): focal immunostaining of TGF-β1 (arrow) (**F**). Moderate staining of scattered cells. inset; (**f**). (**G, g**): focal immunolabeling of TNF-α (**G**). Intensive staining of inflammatory cells inset (**g**). (**H, h**): focal immunoexpression of VEGF (**H**). Intensive staining of cells is presented inset; (**h**). E, G, H = 400 µm, F = 150 µm & (**e**) h = 40 µm. Rosmarinic 70 {**I–L**} (**I, i**): Fibrous connective tissue formation is prominently limited in the area compared to control and Rosmarinic 50 groups (asterisk). The small amount of edema is also obvious at the bottom (blank asterisk) (**I**). Visceral cavity (Vc) Mature scar as dense collagen is present with microvascular channels (arrows) and few inflammatory cells (arrows) inset; (**i**). HE. (**J, j**): low population of cells demonstrates immunolabeling with TGF-β1 (arrows) (**J**). only a few inflammatory cells appear with strong staining inset; (**j**). (**K, k**): mild immunoreaction of TNF-α (**K**). only a few cells demonstrate deep brown staining in their cytoplasm (arrows) inset; (**k**). (**L, l**): scant cells are immunoreacted with VEGF (**L**). A small number of inflammatory cells are deeply immunoreacted (arrows) inset; (l). (**I**) L = 400 µm & i–l = 40 µm.

**Figure 3:**
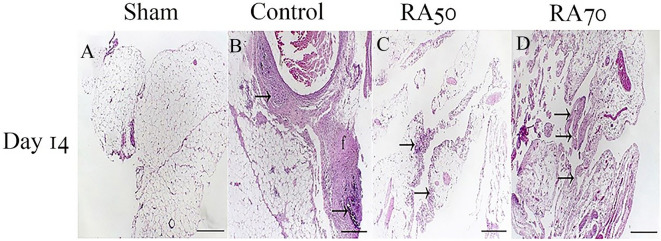
Visceral peritoneum; (**A**) Sham group. (**B**) Control group. Intensive granulomatous inflammation (arrows) and fibroblast proliferation (f). (**C**) Rosmarinic 50 group. Moderate inflammation of the peritoneum, which is mostly bordered at the margins (arrows). (**D**) Rosmarinic 70 group. Mild to moderate fibroblast proliferation with a low population of inflammatory cells throughout the lesion. A–D = 400 µm.

On day 14 after surgery, histological specimens and mean fibroblast proliferation and inflammation scores are shown in Figs. 2 and 3 according to modified Zühlke’s scale. In this research, there was a statistically significant difference in the score of inflammation in terms of chronic inflammatory cells, necrosis, and edema between the treatment (RA50, RA70) and control groups (p < 0.05). Besides, as compared to the control group, the treatment groups showed a significant reduction in fibroblast proliferation (p < 0.05). In terms of the measured scores, however, there was no statistically significant difference among the treatment groups (p > 0.05). Based on Zühlke’s scale, no adhesion was found in the sham group and the score for this group was 0. A significant difference was observed among the control (3.8 ± 0.44), RA 50 (1.6 ± 0.54), and RA 70 (1.4 ± 0.54) groups for the Zühlke grade (p < 0.05). No significant difference was found between RA 50 and RA 70 treatment groups (p > 0.05).

The diagram shows inflammation, fibroblast proliferation, and IHC scores. * Indicates significant difference with the control group.

### Rosmarinic acid reduced postoperative peritoneal adhesion formation by reducing TGF-β1 expression

**Figure 4:**
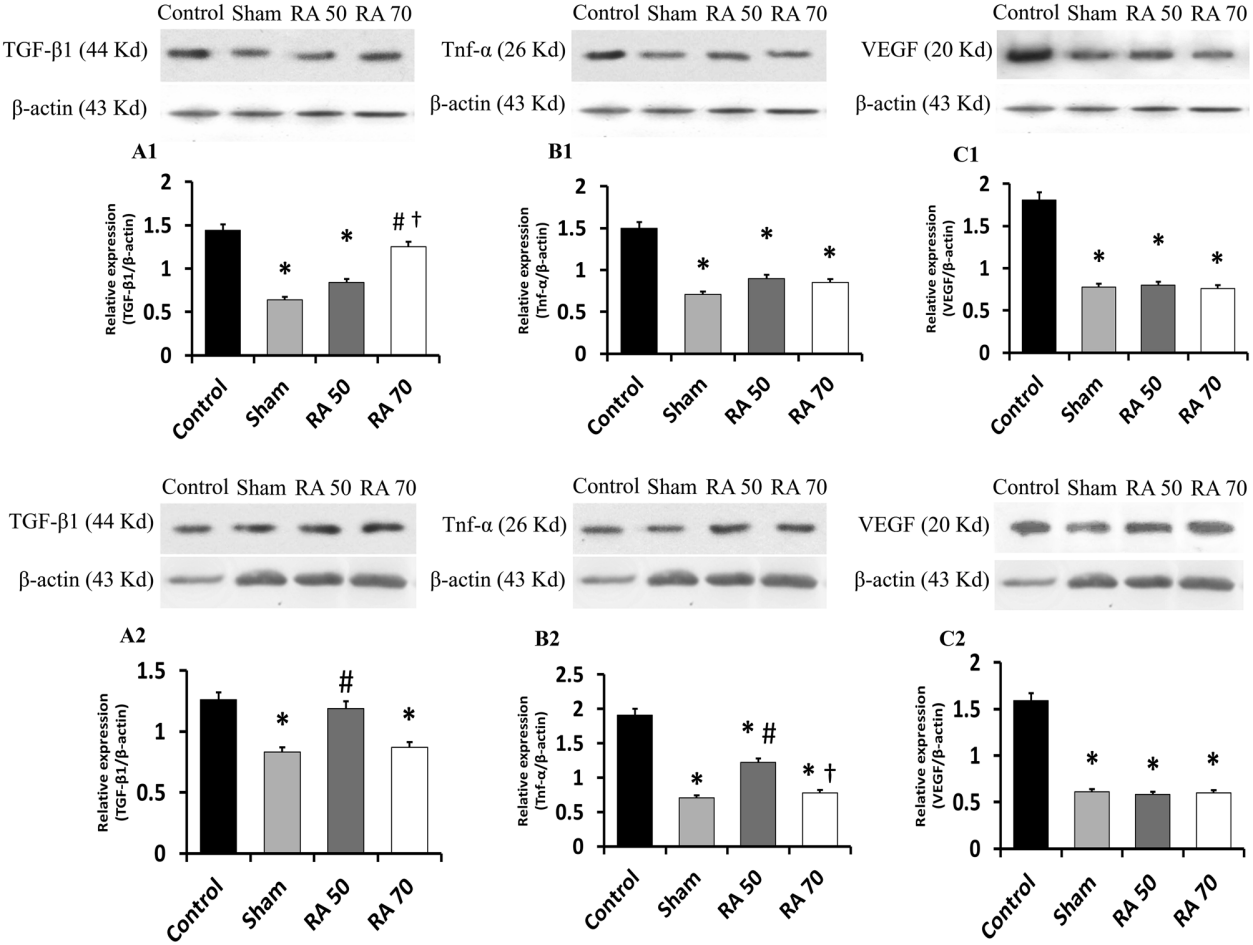
**A1, B1**, and **C1** show western blot analysis for TGF-β1, TNF-α, and VEGF on day 3. **A2, B2,** and **C2** show western blot analysis for TGF-β1, TNF-α, and VEGF on day 14. * Indicates significant difference with the control group. # Shows significant difference with the sham group. † Shows significant difference with the RA 50 group. The photo-micrographs were cropped from the same gel and original blots/gels are presented in Supplementary Figs. 1–8.

On the third day after surgery, western blot analysis revealed that the expression of TGF-β1 in the sham and RA50 groups was significantly lower than in the control group (p < 0.05) (Fig. 4A1). However, as compared to the sham and RA50 groups (p < 0.05), the RA70 group showed a considerable increase but was not statistically different from the control group (p > 0.05). On day 14, the expression of TGF-β1 in the control group was significantly higher than that in the sham group (Fig. 4A2) (p < 0.05). TGF-β1 levels were also considerably lower in the RA70 group than in the control group (p < 0.05). However, the RA50 group showed a significant rise when compared to the sham group (p < 0.05), whereas the control and RA50 groups, showed no significant difference (p > 0.05). TGF-β1 expression in immunohistochemical labeling in the RA50 and RA70 groups compared to the control group on day 14 demonstrated the aforementioned findings, which were also supported by semi-quantification analysis (p < 0.05) (Figs. 2, 3).

### Rosmarinic acid reduced postoperative peritoneal adhesion by inhibiting TNF-α expression

TNF-α expression levels by Western blotting on the third postoperative day are shown in Fig. 4B1. TNF-α levels were considerably lower in both treatment groups compared to the control group (p < 0.05), as seen in the figure. Compared to the sham group, the control group shows a substantial increase (p < 0.05). Rats in both the treatment and sham groups indicated a significant reduction on day 14 after surgery when compared to the control group (p < 0.05) (Fig. 4B2). Furthermore, as compared to the RA50 group, the RA70 group substantially lowered TNF-α levels (p < 0.05). TNF-α levels in the RA50 group, on the other hand, were significantly higher than in the sham group (p < 0.05). Moreover, the immunohistochemical and semi-quantitative analysis demonstrated a substantial reduction in TNF-α levels on day 14 (p < 0.05) as compared to the control group (Figs. 2, 3).

### Rosmarinic acid reduced angiogenesis in postoperative peritoneal adhesion by inhibiting VEGF expression

On days 3 and 14, the levels of VEGF expression are shown in Fig. 4C1 and C2. On days 3 and 14, postoperatively, both doses of rosmarinic acid showed a substantial reduction in VEGF expression compared to the control group (p < 0.05). Furthermore, immunohistochemical and semi-quantitative analysis results on day 14 following surgery were consistent with the aforesaid findings (p < 0.05) (Figs. 2, 3).

### Rosmarinic acid reduced postoperative oxidative stress

**Figure 5:**
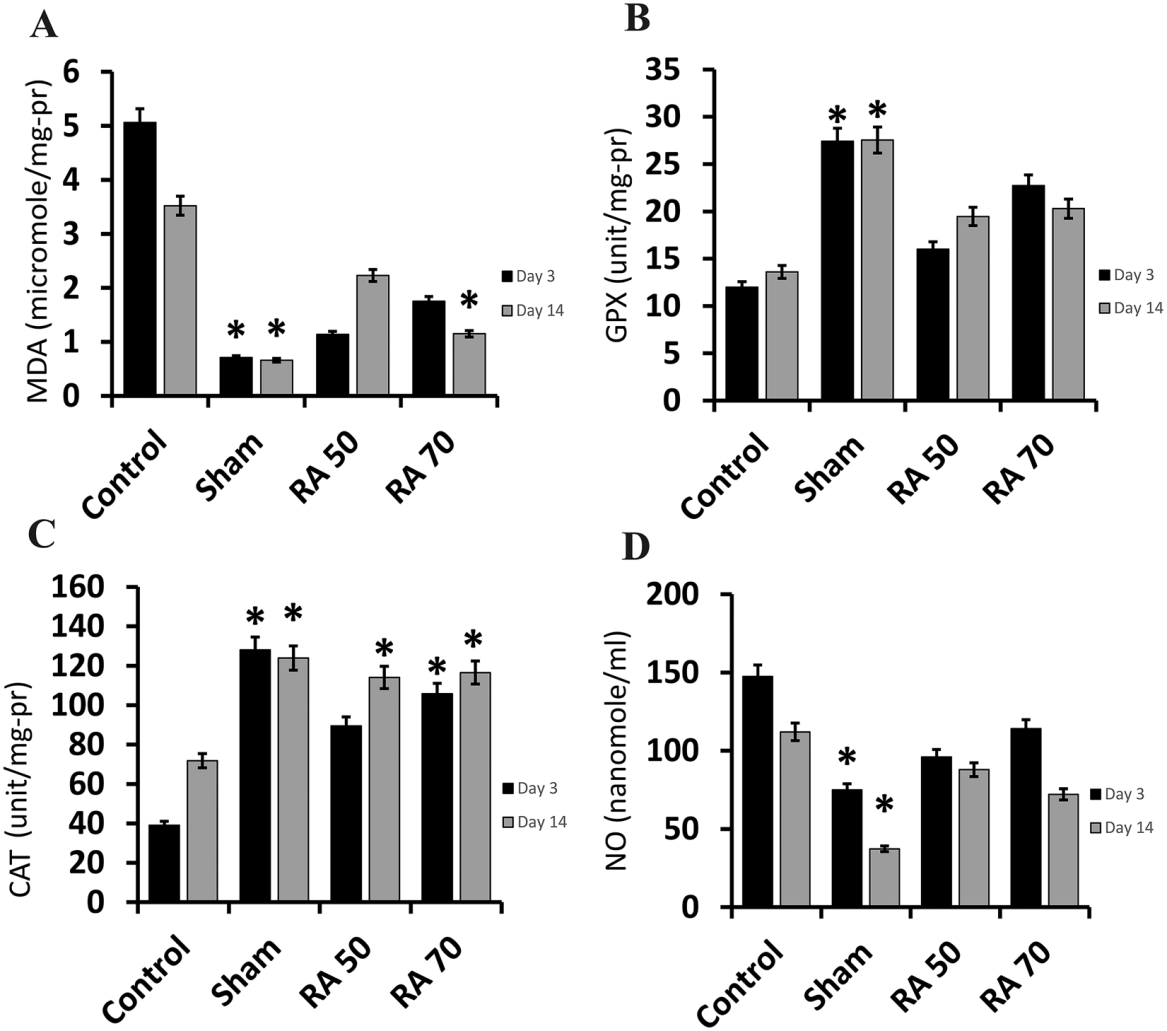
Oxidative stress biomarkers in various experimental groups of the study on days 3 and 14. (**A**) Malondialdehyde (MDA); (**B**) Glutathione peroxidase (GPx); (**C**) catalase (CAT); (**D**) Nitric oxide (NO). * Indicates significant difference with the control group.

On days 3 and 14 after surgery, the levels of oxidative stress indicators in the peritoneal fluid are shown in Fig. 5. On days 3 and 14 after surgery, there was a significant difference between the sham group and the control group in all evaluated biomarkers (p < 0.05). On day 14 after surgery, MDA concentration in the RA70 group was significantly lower than in the control group (p < 0.05). On both days, the decrease in MDA levels in the RA50 group was not significant, and on the third day, it was not significant in the RA70 group (p > 0.05) (Fig. 5A). When compared to the control group, the increase in GPx levels in the treatment groups was not significant (p > 0.05) (Fig. 5B). On both days, rats in the RA70 group, as well as the RA50 treated group on day 14, had significantly higher CAT levels than the control group (p < 0.05). However, as compared to the control group, the increase in CAT level in the RA50 group on day 3 was not significant (p > 0.05) (Fig. 5C). NO levels in the treatment groups decreased, although not significantly (p > 0.05) compared to the control group (Fig. 5D).

## Discussion

**Figure 6:**
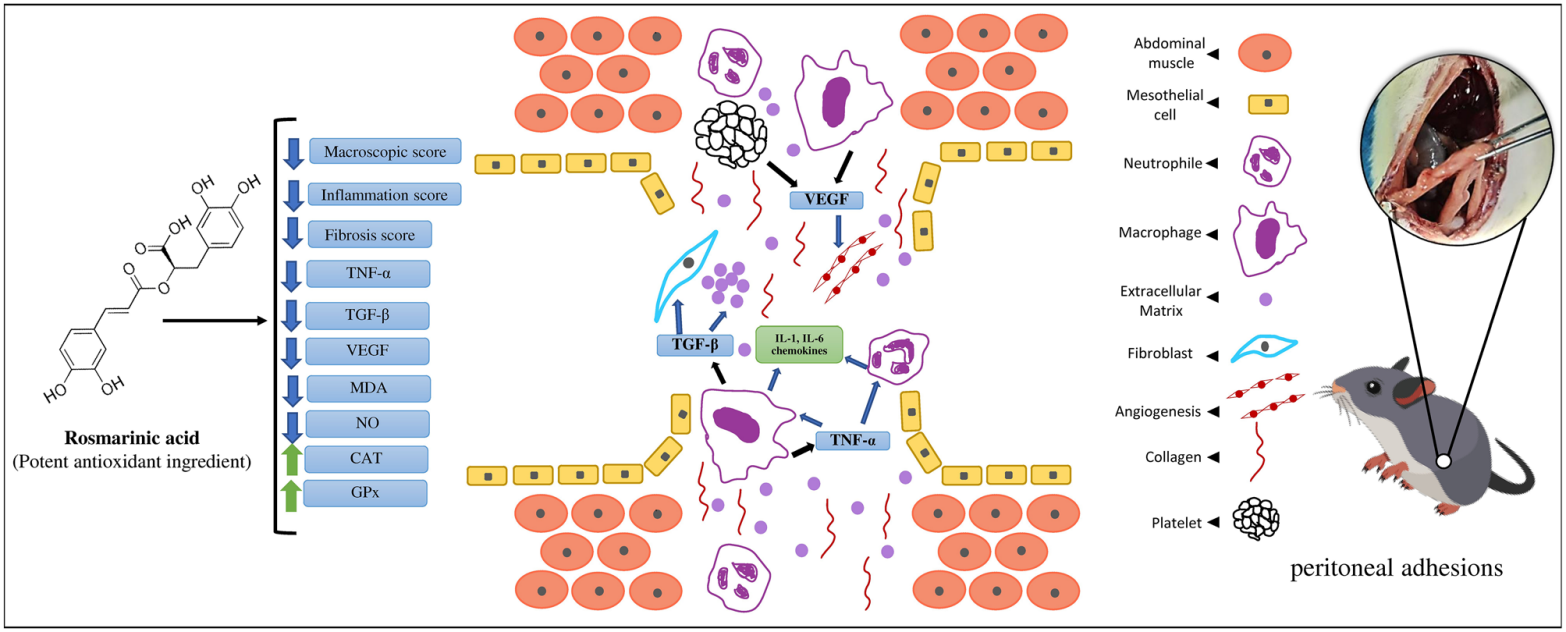
Rosmarinic acid reduced the development of postoperative intra-abdominal adhesion. Rosmarinic acid decreased inflammation, fibroblast proliferation, and inflammatory cytokines such as TGF-β1, TNF-α, and VEGF. Moreover, rosmarinic acid reduced oxidative stress. As a result, collagen deposition, angiogenesis, secretion of extracellular matrix (ECM), and fibroblast production are reduced, which are all involved mechanisms in adhesion development.

Following the surgical procedures in the abdominal cavity, peritoneal adhesion formation is always a concern which occurs in approximately 93% of patients experiencing a laparotomy^32^. Excessive manipulation during surgery, damage to the peritoneal cavity, and the entry of infectious organisms into the abdomen, which induces fibrin deposition, all produce peritoneal adhesions^33^. Inflammatory responses and the admission of inflammatory cells into the region, overproduction of reactive oxygen species, and fibrin deposition all occur concurrently with the onset of healing in the peritoneum, all of which are factors in the creation of adhesive bands (Fig. 6)^34^. Peritoneal adhesions are still one of the most prevalent consequences of abdominal surgery, despite major advancements in laparotomy operations and the advent of less invasive methods^32^. Previous research showed that the healing process begins the day after mesothelial cell injury and takes 8–10 days to complete. Peritoneal incisions and the removal of a small piece of the peritoneal layer were used to generate the experimental model in this work^35,36^. On days 3 and 14 after surgery, the samples were obtained to assess the chronic and acute phases of the adhesion process.

In previous studies, macroscopic scores were widely utilized to analyze the establishment of adhesions. On days 3 and 7, the effects of peripheral serotonin on postoperative intra-abdominal adhesion development were examined, and the findings showed that Tryptophan hydroxylase 1 knockout mice exhibited lesser adhesion bands than wild-type rats^37^. Adhesion scores were assessed on day 14 post-surgery in another research by Askari et al., and groups treated with Iranian propolis had lower adhesion ratings than the control group^38^. On days 3 and 14, the macroscopic scores of the peritoneal adhesions were assessed using the grading technique published by Nair et al.^39^. On day 3, there were few adhesion bands, and there was no significant difference between groups, according to the findings. On day 14, however, treatment groups had a considerably lower adhesion score than the control group.

After peritoneal damage, inflammation is common, leading to the release of inflammatory cytokines and the development of fibrous bands^40,41^. Previous research^42^ showed the anti-inflammatory benefits of rosmarinic acid in inflammatory disorders such as colitis, arthritis, and atopic dermatitis. In a research, Rocha et al. surveyed the anti-inflammatory effects of rosmarinic acid in rat models of local and systemic inflammation and discovered that RA dramatically decreased inflammation following thermal damage^43^. Previous research has shown that RA has antithrombotic and fibrinolytic properties^44^. Inflammation and fibroblast proliferation were considerably lower in the RA-treated groups compared to the control group, confirming earlier findings.

TGF-β1 is a pleiotropic cytokine that helps with wound healing, angiogenesis, and immunoregulation^45^. It may either suppress or reduce inflammation, which is a double-edged sword. The presence of IL-6 on leukocytes has a strong chemotactic impact in the early stages of tissue damage, resulting in a fast accumulation of macrophages and PMN^46,47^. As a result, the immunosuppressive effect is dominant when combined with IL-2, which enhances FOXP3 expression and T-regulatory cell clonal development. T-helper 1 and 2 cells, NK cells, PMNs, CTLs, macrophages, and DCs are among the immune cells impacted, highlighting the bipolar character of the disease^48,49^. TGF-β1 enhances the production of other cytokines such as VEGF and CTGF in late stages of inflammation, which contribute to angiogenesis and fibrosis while suppressing the production of pro-inflammatory cytokines, such as MIP-α1, IL-1β, TNF-α, and IFN-γ^45,50^. TGF-β1 and fibrosis grade were considerably reduced in an experimental liver fibrosis rat model treated with RA^24^. Lin et al. studied the effects of rosmarinic acid in an extrahepatic cholestasis rat model and found that TGF-β1 expression in the liver tissue of the treatment groups was considerably decreased^51^. Ghadiri et al. surveyed the effects of pomegranate peel extract lavage on peritoneal adhesion and found that TGF-β1 expression was significantly reduced in the extract-treated group^52^. In line with prior research, TGF-β1 expression in IHC stained slides was dramatically decreased in the RA 70 group. On days 3 and 14, relative expression of TGF-β1/β-actin in the treatment groups was considerably lower than in the control group.

When the peritoneal damage occurs, inflammatory cells, such as neutrophils, monocytes, and lymphocytes migrate to the injured area. TNF-α is mainly secreted by monocytes which are regarded as one of the principal inflammatory cytokines which identify the degree of adhesion^2^. Rocha et al. surveyed the anti-inflammatory impact of rosmarinic acid on thermal damage and found that rosmarinic acid considerably lowered blood TNF-α levels when compared to the control group^43^. Rosmarinic acid was shown to be efficient in lowering TNF-α expression in a mouse model of acetaminophen-induced liver injury in a study^53^. TNF-α levels in the treatment groups were considerably lower on day 3 compared to the control group. TNF-α expression was considerably lower in the treatment groups on day 14, with RA 70 much lower than the RA 50 group. The expression of TNF-α in the IHC stained slides matched the western blot results.

The formation of adhesion bands is complicated by angiogenesis. As a consequence of hypoxia in the injured peritoneum, macrophages produce vascular endothelial growth factors to deliver blood, oxygen, and nutrients for the healing process^54^. Adhesion formation may be reduced by substances that inhibit VEGF expression and angiogenesis^55^. In research by Huang et al., rosmarinic acid inhibited the proliferation and migration of human umbilical vein endothelial cells. Furthermore, rosmarinic acid reduced VEGF-positive immunohistochemistry cells^17^. The protective effects of rosmarinic acid on hepatocellular carcinoma inflammation and angiogenesis were surveyed, and the findings indicated that RA significantly decreased VEGF^29^. In a rat model of ethanol-induced gastric ulcer, rosmarinic acid decreased VEGF immunopositive cells^56^. In an ovarian torsion/detorsion rat model, rosmarinic acid therapy decreased VEGF IHC expression in ovarian cells^57^. These findings support our findings, indicating that rosmarinic acid dramatically reduces VEGF expression on days 3 and 14 in both western blot and IHC.

Hypoxia develops at the site of peritoneal damage, which is followed by the release of reactive oxygen species and oxidative stress^58^. Inflammation and tissue hypoxia causes mitochondrial malfunction, resulting in an increase in reactive oxygen and nitrogen species in the injured tissue. In order to scavenge and neutralize free radicals, the amount of antioxidant enzymes in the damaged area rises^58^. Sadeghi et al. found that rosmarinic acid lowered MDA and NO levels while increasing the activity of GPx and SOD enzymes in lipopolysaccharide-induced peripheral blood mononuclear cells^59^. Another study found that rosmarinic acid protects cells from oxidative damage by lowering MDA levels and increasing SOD levels in acrylamide-induced cell death^60^. The amount of SOD and CAT in the liver and kidney of diabetic rats was reduced by rosmarinic acid^61^. The findings of this study support earlier observational studies that show rosmarinic acid decreases oxidative stress by lowering MDA and NO levels while increasing GPx and CAT levels.

## Conclusion

This study aimed to survey whether rosmarinic acid may help prevent post-operative abdominal adhesions. Our data show that rosmarinic acid inhibited peritoneal adhesion, inflammation, and fibroblast proliferation, as well as the release of inflammatory cytokines including TGF-β1 and TNF-α. It also inhibited angiogenesis. Furthermore, antioxidant actions such as reducing MDA and NO while raising GPx and CAT levels contribute to its positive benefits. The present research was conducted just on rats. Future research in other species will be necessary to assess deeper processes. Furthermore, clinical research on the effects of rosmarinic acid on peritoneal adhesions should be conducted.

## Materials and methods

### Animal studies

40 healthy male Wistar albino rats weighing nearly 250 to 300 g and aged 6 to 8 weeks that had not previously undergone previous surgical or any medical intervention were obtained. They had not previously experienced any surgical or medicinal intervention. The animals were housed in the Lorestan University of Medical Sciences animal house under conventional settings, which included a 12-h light cycle, 65% ± 3% humidity, and a temperature of 23 ± 2 °C, as well as unrestricted access to plate food for laboratory animals and water. The current research was reviewed and approved by the Lorestan University ethics committee (code NO. LU. ECRA.2022.15, Lorestan University, Faculty of Veterinary Medicine). All methods were performed in accordance with the relevant guidelines and regulations. This study was carried out in compliance with ARRIVE's guidelines.

The rats (n = 40) were split into four groups of ten rats each at random:

- Sham group: The induction of anesthesia and midline surgical incision without the induction of adhesions (n = 10).
- Control group: The induction of adhesion and treatment with 3 ml of normal saline (n = 10).
- Rosmarinic acid 50 mg/kg (RA 50) group: The induction of adhesion and treatment with 3 ml of rosmarinic acid (SIGMA-ALDRICH, CO.,3050 Spruce Street, St. Louis, MO 63178 USA 314-771-5765) at a dose of 50 mg/kg^62^ (n = 10).
- Rosmarinic acid 70 mg/kg (RA 70) group: The induction of adhesion and treatment with 3 ml of rosmarinic acid at a dose of 70 mg/kg^62^ (n = 10).

None of the animals in this study were excluded from analysis and experiments. Moreover, no deaths or major complications were found during the experiments.

### Surgical procedure

In all groups, the anesthesia was intramuscularly administered with a mixture of ketamine and xylazine (75 mg/kg / 10 mg/kg). Because the rats were operated on under sterile settings, no antibiotics were administered to them. During the procedure, the breathing and heart rate of animals were monitored. After the anesthesia of rats, for inducing the adhesions in experimental groups, the hair of the surgical site was shaved and the skin of the abdomen was disinfected by applying 10% Povidone-iodine solution. Subsequently, a 3 cm long incision was created in the abdomen's midline. Except for the sham group, 3 shallow incisions of 2 cm were made longitudinally and transversely in the parietal peritoneum of the right abdominal wall after entering the abdominal region in the other groups. Then, on the left side of the inner wall of the abdomen, a portion measuring 1 × 1 cm was cut from the peritoneal surface using a surgical scissor. Immediately after the development of lesions, 3 ml of the required solution was poured over the lesion site in RA 50 and RA 70 treatment groups. A positive control group was formed by pouring 3 mL of normal saline over the injured area. The sham group of rats did not have any lesion inductions and did not get any treatments; hence they were deemed a negative control. Suture material 3–0 polyglycolic acid (PGA, Supa, Karaj, Iran) was used to stitch linea alba and 4–0 Polyamide suture material was used to seal the abdomen (Nylon; Ethicon Inc., Somerville, NJ). Finally, the rats' skin was sutured, and they were placed in temperature-controlled environments until they recovered consciousness. The first day of therapy was the day when adhesions were induced.

### Macroscopic evaluation and grading of adhesions

Each group of animals was separated into two equal groups with half of them undergoing reoperation after three days and the other half after fourteen days of surgical procedures. Adhesion grading was conducted by a person who was ignorant of the groups after the anesthesia and opening the abdomen of each rat using the procedure provided by Nair et al.^39^. An overdose of thiopental sodium (250 mg/kg) was used to euthanize the rats.

### Sample collection method

To analyze the oxidative stress indicators, peritoneal fluid samples were taken by lavaging the peritoneal cavity with 2 mL of sterile saline and then maintained at − 80°C^63^. The adhesive bands as well as the underlying tissues were sampled, sliced equally, and cleaned with an aseptic PBS solution. For Western blotting, half of the tissues were maintained at – 80 °C, while the other half were deposited in a 10% neutral buffered formalin solution for histopathological and immunohistochemical examination.

### Histopathological evaluation

Tissue samples, including the peritoneal adhesions and underlying tissue (abdominal muscles), were fixed in a 10% neutral buffered formalin solution. The next stages for the H&E staining method were performed as follows. Dehydration by applying the ethylene alcohol, clearing using xylene, and preparing blocks by embedding them in paraffin wax were done, respectively. Afterwards, 3 µm pieces were made using a rotary microtome. Eventually, H&E staining was performed and inflammation and fibroblast proliferation scores were done in three degrees based on our previous study^34^. A Pathologist who was blinded to the groups evaluated the H&E slides and performed Zühlke’s microscopic adhesion classification^64^.

### Immunohistochemical evaluation

Immunohistochemistry staining on peritoneal sections was conducted using the following antibodies: polyclonal rabbit anti-TGF-b (MBS462142, Mybiosource, USA) at 1:100 dilution, TNF- (ab6671, Abcam, UK) at 1:100 dilution, and VEGF (LS-B7747, LSBio, USA) at 1:100 dilution in the current investigation. The sections were deparaffinized, rehydrated, and treated in a target retrieval solution of Tris–EDTA (pH = 9). To deliver unmasked antigens, samples containing the target retrieval solution were put in a 98 °C heating bath and kept there for 20 min. To halt endogenous peroxidase, samples were treated with hydrogen peroxide (3 percent H2O2) in PBS for 15 min, and normal rabbit serum (5 percent) in PBS was used to avoid nonspecific background staining. The samples were incubated with primary antibodies for one hour. Secondary antibody staining using a goat antirabbit biotinylated antibody (prediluted, Biocare, USA) for 20 min was used to identify primary antibodies. Then, 20-min incubation with prediluted streptavidin horseradish peroxidase (sHRP) (Biocare, USA) was done. Finally, DAB was used as a chromogen to observe antibody binding areas, and Mayer's Hematoxylin (Bio Optica, Italy) was used to counterstain the samples. IHC grading was performed on a scale of 0 to 3 (0: no reaction ǀ 1: mild reaction ≤ 10% ǀ 2: moderate reaction 10–30% ǀ 3: intensive reaction ≥ 30%).

### Western Blotting

Previous studies^65^ were used to guide the western blot. To reduce tissue proteases and facilitate protein extraction, adhesion tissue samples were first homogenized in RIPA lysis buffer solution (Sigma-Aldrich S8820, USA). Then, a loading buffer was used to dilute the protein samples, which were then heated in a 95 °C bath for five minutes. Then, the isolated proteins were electrophoresed on a polyvinylidene fluoride membrane (PVDF) at 100 V for 1–2 h using sodium dodecyl sulphate–polyacrylamide (SDS-PAGE, 120 V). PVDF membrane was treated in a particular buffer (5 percent non-fat milk) for a night to suppress endogenous peroxidases. Hence, the membranes were washed in Tris-buffered saline (pH = 7.2, including 0.1 percent Tween 20, × 3, 15 min each time) and incubated with TNF-α, TGF-β1, VEGF, and β-Actin (E-AB-40015, E-AB-67255, E-AB-33090, E-AB-20058) antibodies for two hours at 4 °C. Unattached antibodies were rinsed with a washing solution before being incubated for 60 min at 25 °C with horseradish peroxidase (HRP)-conjugated secondary antibody (E-AB-1003, E-AB-1001). Finally, an enhanced chemiluminescence detection kit (ECL, Thermo Scientific, USA) was used to visualize the blots. Using improved laser densitometer software, the relative intensity of protein/β-Actin was determined (Arash-Teb-Pishro, Iran).

### Oxidative stress biomarkers analysis

Commercial biochemical kits (Asan, Khorramabad, Iran) were used to measure the malondialdehyde (MDA), glutathione peroxidase (GPx), catalase (CAT), and total antioxidant capacity (TAC) levels in the peritoneal fluid based on manufacturer's procedure.

### Malondialdehyde (MDA)

The biomarker malondialdehyde is used to identify lipid peroxidation and cell damage. Previous studies^66^ showed that the thiobarbituric acid (TBA) approach is an accurate method to detect MDA. Kit methods were followed to evaluate MDA levels in peritoneal fluid samples. Initially, 100 μL of the sample was put into a tube. The tube was then filled with 1500 μL TBA 0.06 percent and 1000 μL TCA 1 percent solutions. Tubes were submerged in a 95 °C water bath for 30 min. Then, the supernatant was centrifuged at 1000 rpm for 15 min, and the absorbance of the supernatant was measured using a spectrophotometer (JENWAY 6715 UV/Vis Spectrophotometer, Staffordshire, UK). The MDA concentration in peritoneal fluid samples was measured at μmol/mg-pr.

### Glutathione peroxidase (GPx)

The level of glutathione peroxidase was determined using Assay kit's instructions. All of the chemicals were measured out and placed into a test tube, which was then filled with 200 μl of peritoneal fluid. Thus, the tubes were put in a 37 °C heating bath for 10 min. After that, a three-minute centrifugation at 3000-rpm was performed after 400 μl of 10% tricarboxylic acid was added (TCA). After centrifugation, 25 μl of supernatant were mixed with 140 μl of Tris EDTA solution. The obtained solution was then combined with 30 μL of a 5,5′-dithiobis (DTNB) solution. The resultant solution was eventually kept at 25 °C for half an hour. An ELAISA reader was used to measure the intensity of the solution color at 450 nm. The final GPx values were given in unit/mg-pr.

### Catalase (CAT)

Assan kit protocol to assess the catalase level in the peritoneal fluid was based on the dichromate method described by Sinha^67^. In brief, 50 μl of peritoneal fluid was mixed with 1000 μl of Potassium Phosphate Buffer (PPB, pH = 7). Then, 50 μl of H2O2 was combined with the obtained solution. The absorbance of the resulting solution was measured at 240 nm using a spectrophotometer. Catalase activity in the peritoneal fluid was expressed as unit/mg-pr.

### Nitric oxide (NO)

NO levels in the peritoneal fluid were measured applying Griess' method^68^. NO3 should first be converted to NO2, after which the created NO2 will be determined. The operation was done by mixing 50 μL of peritoneal fluid with 100 μL of reagent (Griess reagent solution). Then, the produced mixture was poured into a 100 mL flask and kept at 25° for 10 min. The absorbance of the samples was measured between 492 and 630 nm. The final NO concentration was given as nanomole/ml.

### Statistical analysis

An investigator who was blind to group allocation and treatments conducted the data analysis. SPSS software was used to examine the data (version 25 for windows, SPSS Inc. Chicago IL USA). Distributions of the groups were analyzed with the one-sample Kolmogorov–Smirnov test. Normally distributed data were expressed as mean ± standard deviation (SD) and non-normally distributed data were expressed as median and interquartile ranges. The data with normal distribution were analyzed using a one-way ANOVA with a Tukey post hoc test. A non-parametric Kruskal–Wallis test was used to compare groups having non-normal distributions. The significance threshold was set at p < 0.05.

## Supplementary Information

Supplementary Information.

## Data Availability

The datasets analyzed during this study are available from the corresponding author on reasonable request.
