# Supplementary material for: Rosmarinic acid prevents post-operative abdominal adhesions in a rat model
Source: Sci Rep. 2022 Nov 3;12:18593. doi: 10.1038/s41598-022-22000-x (PMC9633689; doi:10.1038/s41598-022-22000-x)
Supplement: Supplementary file 1 — Supplementary Information. [file 41598_2022_22000_MOESM1_ESM.doc]

Supporting Information to

**Rosmarinic acid prevents post-operative abdominal adhesions in a rat model**

Ali Kakanezhadi^1†^, Mehrdad Rezaei^1†^, Abbas Raisi^1*^, Omid Dezfoulian^2^, Farshid Davoodi^3^, Hassan Ahmadvand^4^

^1^ Department of Clinical Sciences, Faculty of Veterinary Medicine, Lorestan University, Khorramabad, Iran.

^2^ Department of Pathobiology, Faculty of Veterinary Medicine, Lorestan University, Khorramabad, Iran.

^3^ Department of Surgery and Diagnostic Imaging, Faculty of Veterinary Medicine, Urmia University, Urmia, Iran.

^4^ Medicinal Plants and Natural Products Research Center, Hamadan University of Medical Sciences, Hamadan, Iran.

†Ali Kakanezhadi and Mehrdad Rezaei contributed equally to this work.

* Correspondence: Associate Professor of veterinary surgery, Department of Clinical Sciences, Faculty of Veterinary Medicine, Lorestan University, Khorramabad, Iran.

Email: [raisi.a@lu.ac.ir](mailto:raisi.a@lu.ac.ir); [dr_abbas_raisi@yahoo.com](mailto:dr_abbas_raisi@yahoo.com)

**Original images:**


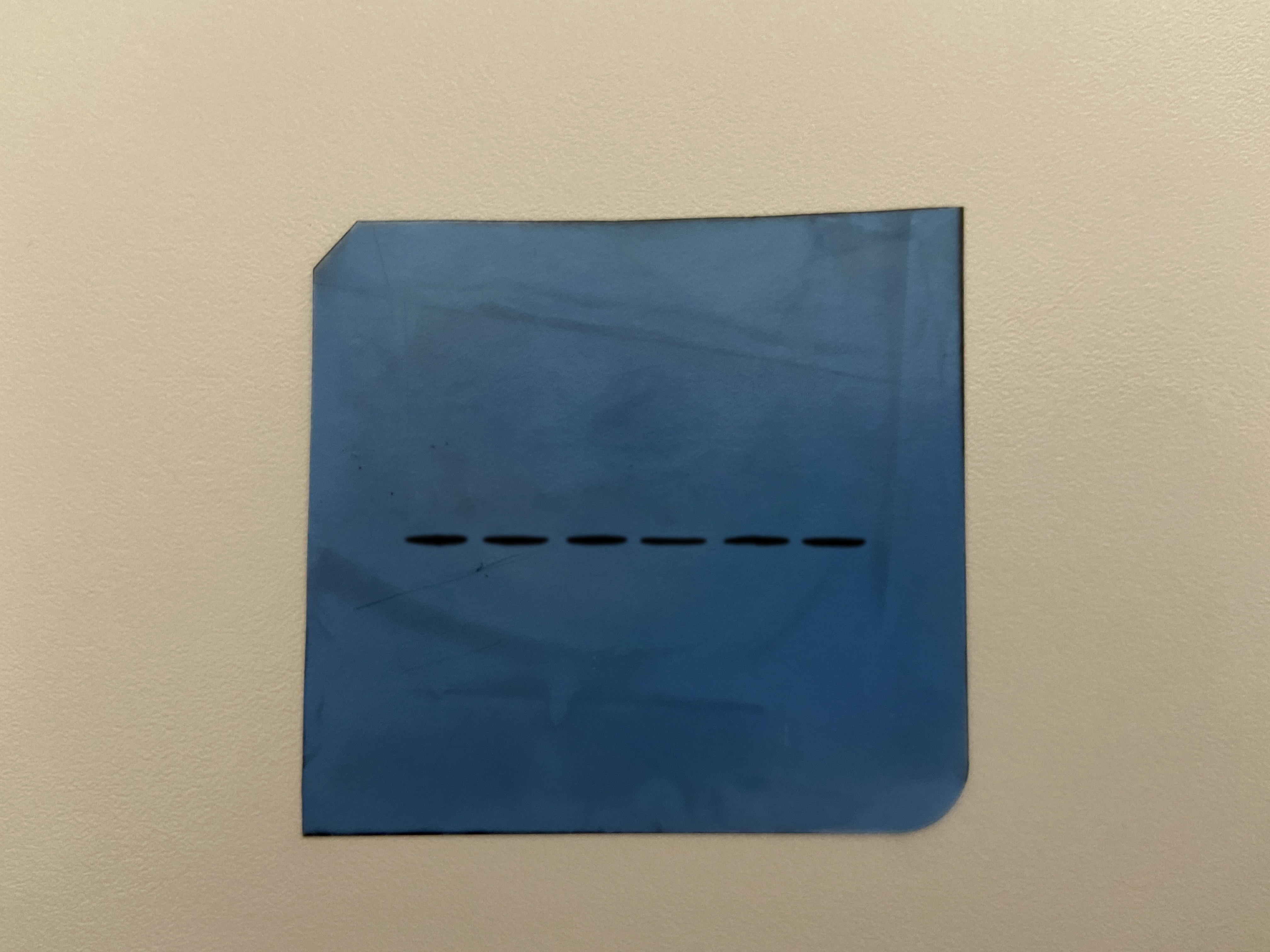


43 KD

Figure 4: Full length gel of western blot for β-actin on day 3 (Four first blots from the left are related to this work respectively).


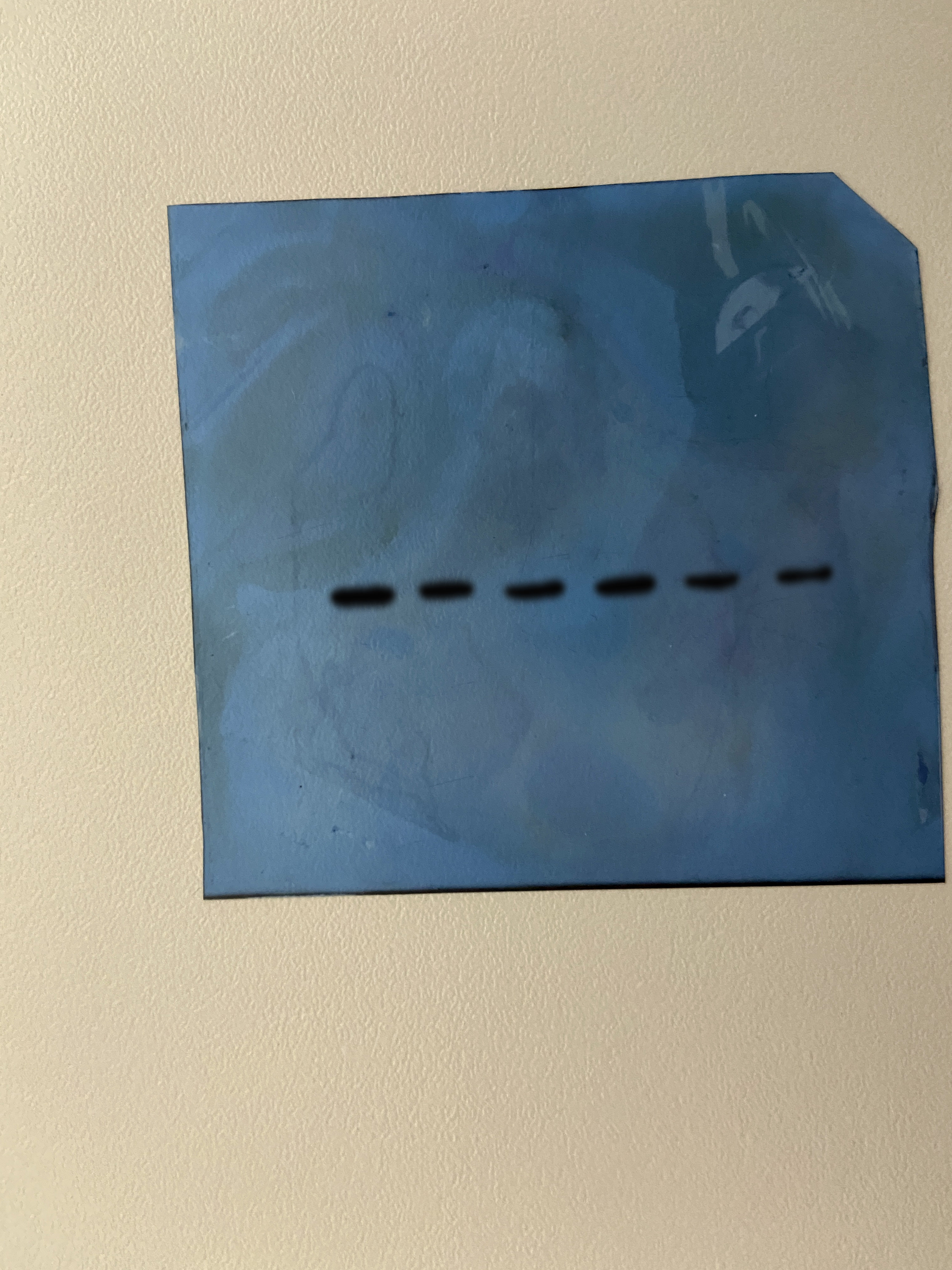


44 KD

Figure 4: Full length gel of western blot for TGF- β1 on day 3 (Four first blots from the left are related to this work respectively).


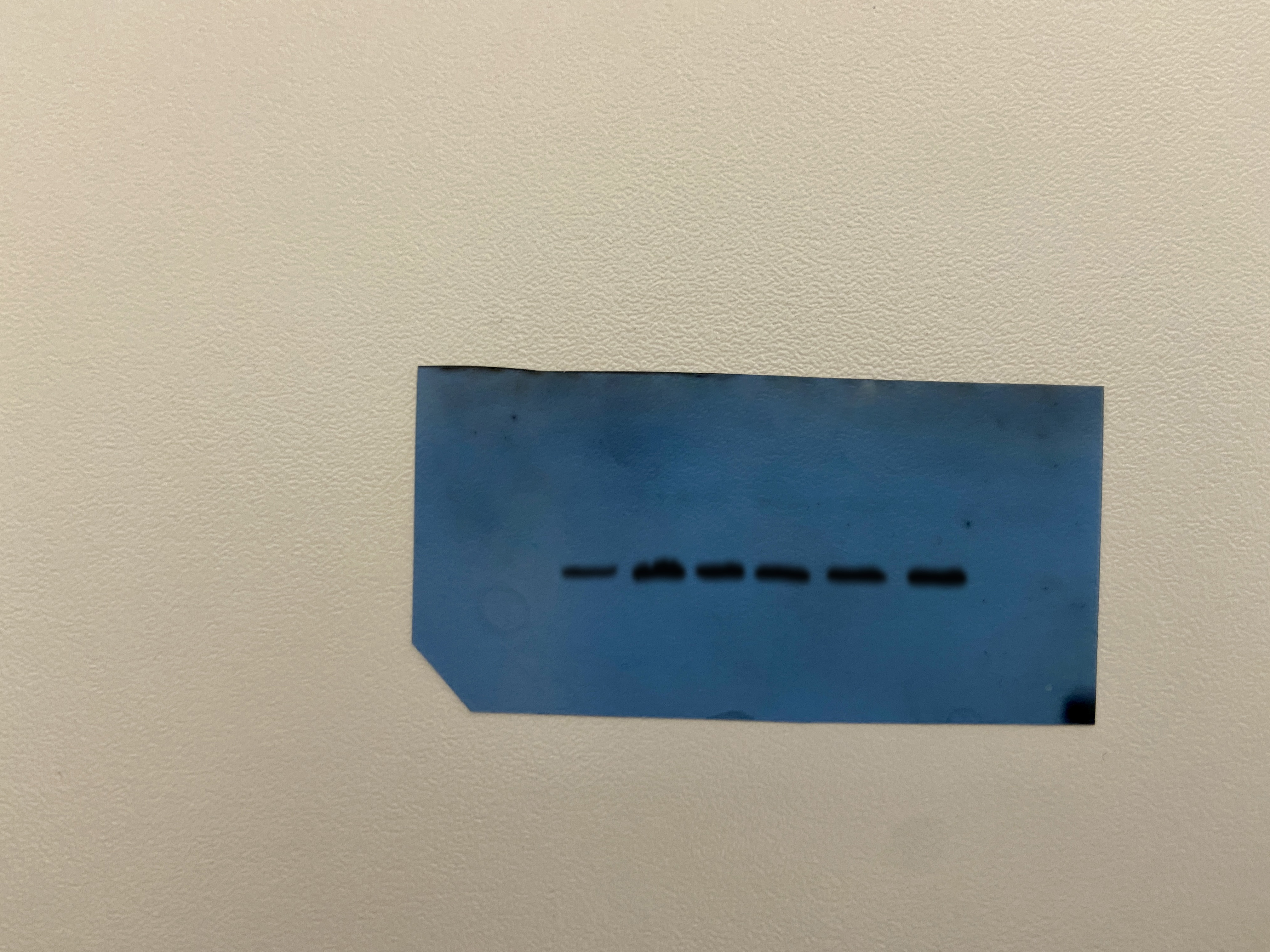


43 KD

Figure 4: Full length gel of western blot for β-actin on day 14 (Four first blots from the left are related to this work respectively).


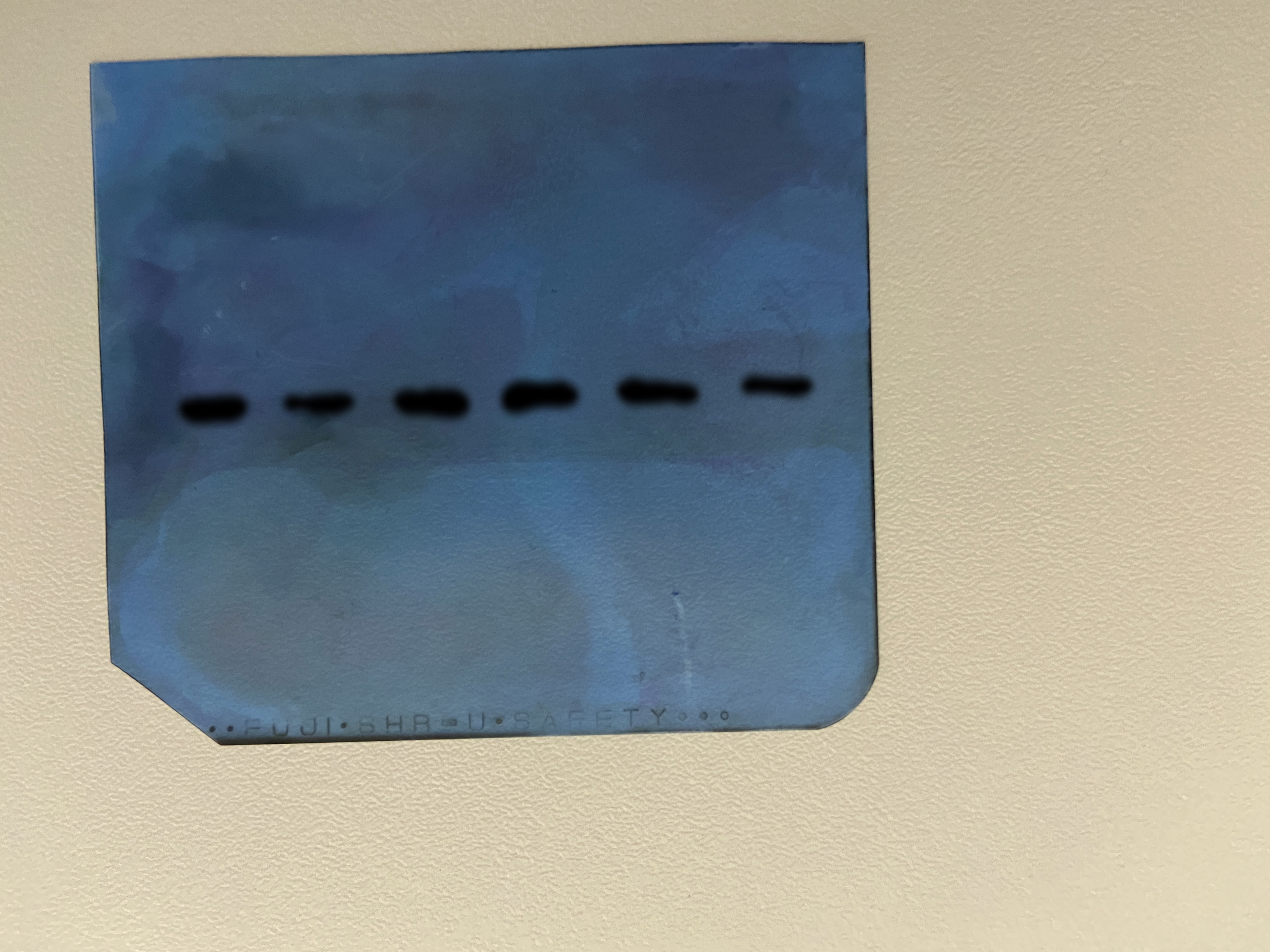


44 KD

Figure 4: Full length gel of western blot for TGF- β1 on day 14 (Four first blots from the left are related to this work respectively).


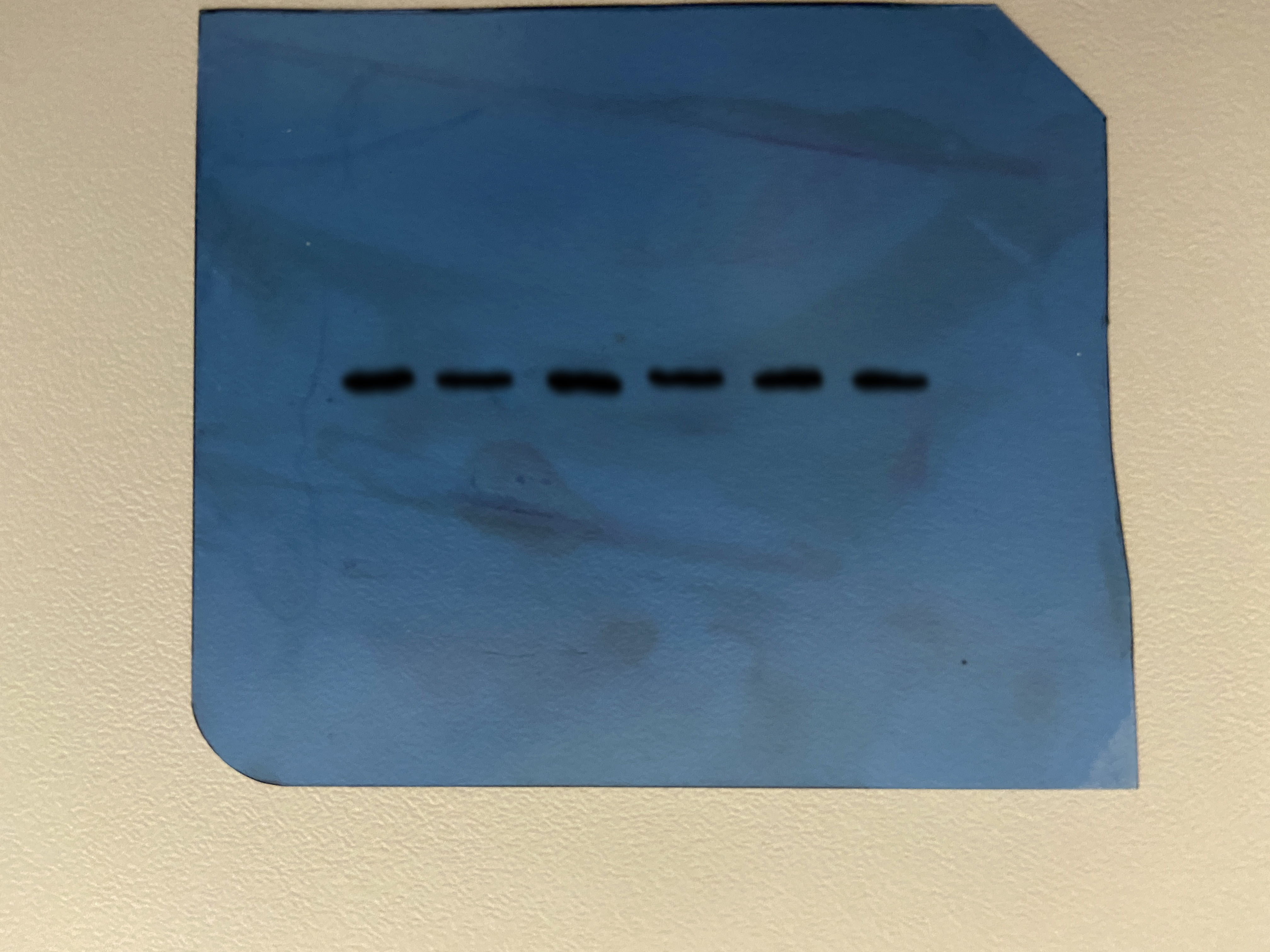


26 KD

Figure 4: Full length gel of western blot for TNF-α on day 3 (Four first blots from the left are related to this work respectively).


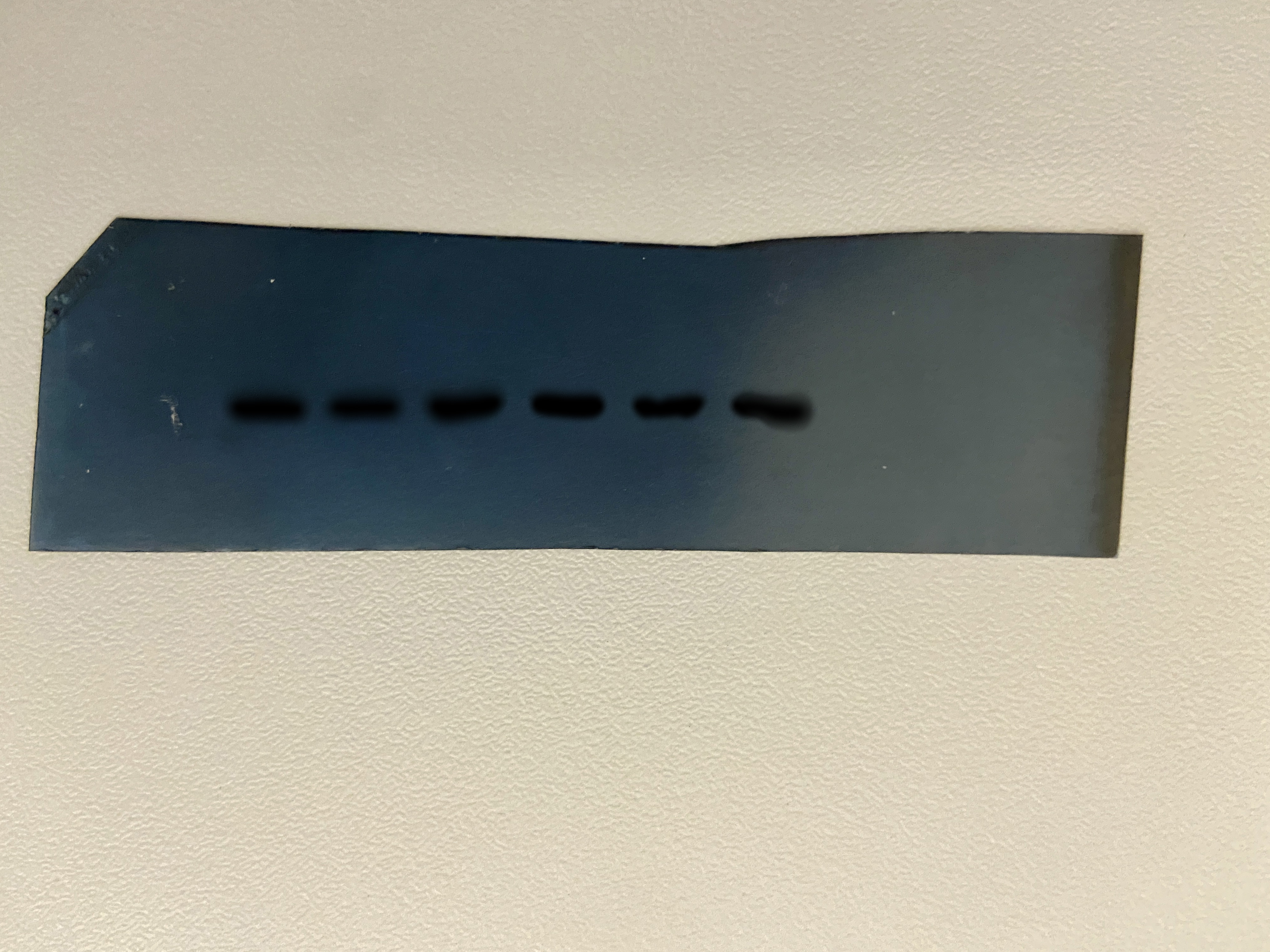


26 KD

Figure 4: Full length gel of western blot for TNF-α on day 14 (Four first blots from the left are related to this work respectively).


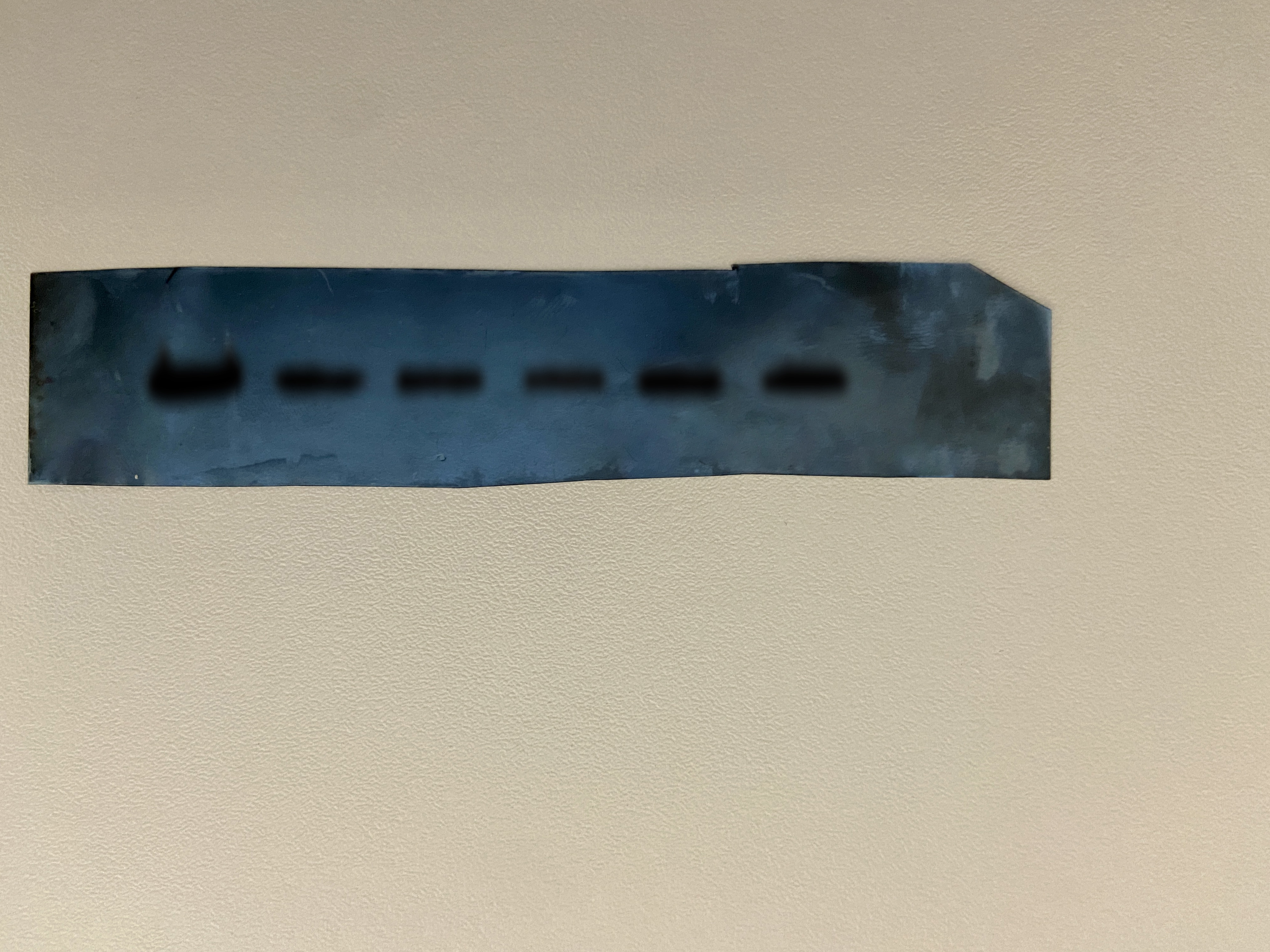


20 KD

Figure 4: Full length gel of western blot for VEGF on day 3 (Four first blots from the left are related to this work respectively).


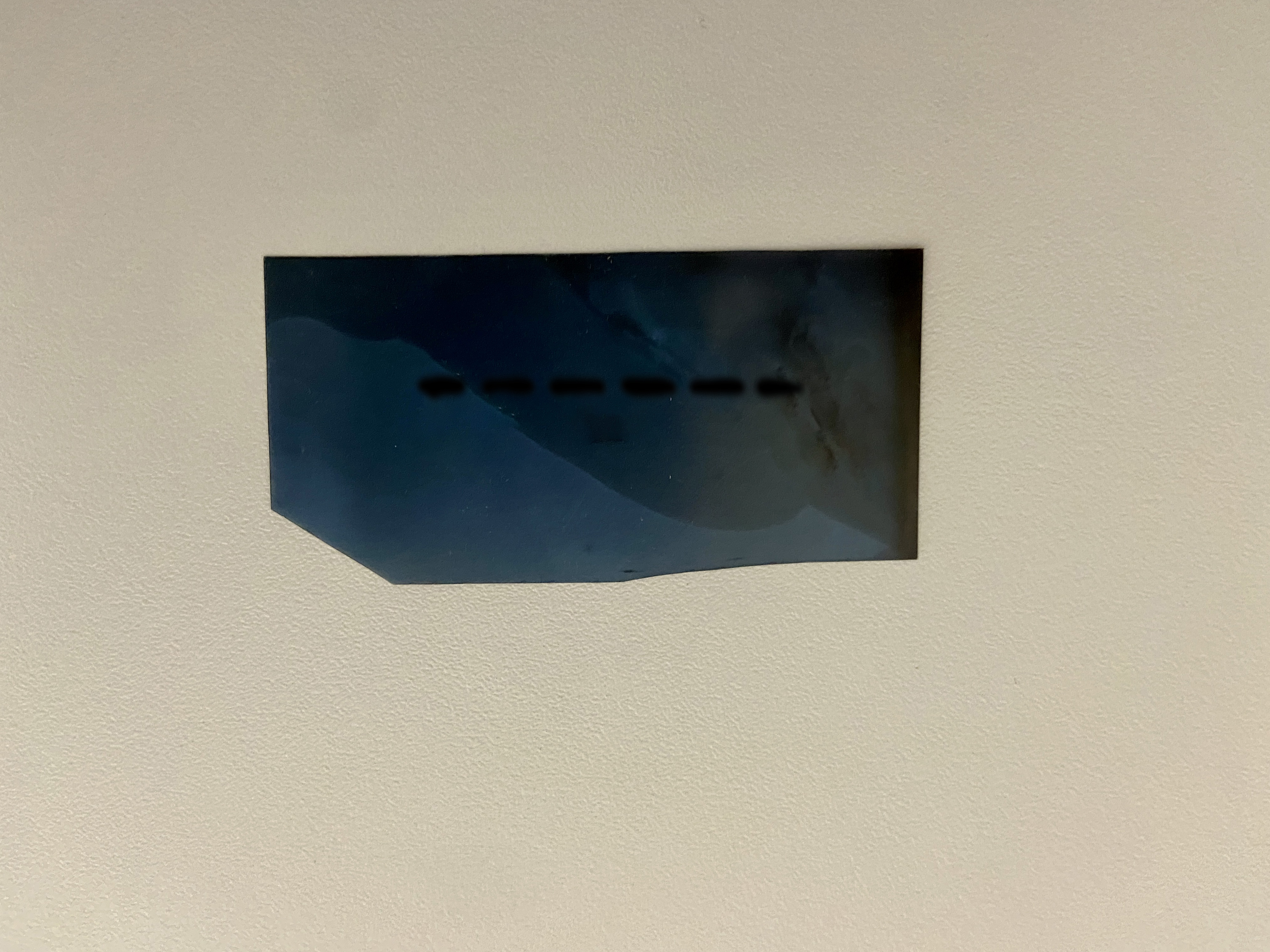


20 KD

Figure 4: Full length gel of western blot for VEGF on day 14 (Four first blots from the left are related to this work respectively).
